# Molecular and Cellular Functions of the Linker Histone H1.2

**DOI:** 10.3389/fcell.2021.773195

**Published:** 2022-01-11

**Authors:** Shuting Lai, Jin Jia, Xiaoyu Cao, Ping-Kun Zhou, Shanshan Gao

**Affiliations:** ^1^ Institute for Environmental Medicine and Radiation Hygiene, School of Public Health, University of South China, Hengyang, China; ^2^ Beijing Key Laboratory for Radiobiology, Department of Radiation Biology, Beijing Institute of Radiation Medicine, Beijing, China; ^3^ School of Medicine, University of South China, Hengyang, China; ^4^ School of Life Sciences, Hebei University, Baoding, China

**Keywords:** linker histone H1.2, DNA damage response, post-translational modification, gene expression, cancer

## Abstract

Linker histone H1.2, which belongs to the linker histone family H1, plays a crucial role in the maintenance of the stable higher-order structures of chromatin and nucleosomes. As a critical part of chromatin structure, H1.2 has an important function in regulating chromatin dynamics and participates in multiple other cellular processes as well. Recent work has also shown that linker histone H1.2 regulates the transcription levels of certain target genes and affects different processes as well, such as cancer cell growth and migration, DNA duplication and DNA repair. The present work briefly summarizes the current knowledge of linker histone H1.2 modifications. Further, we also discuss the roles of linker histone H1.2 in the maintenance of genome stability, apoptosis, cell cycle regulation, and its association with disease.

## Introduction

The genomic DNA (gDNA) in eukaryotes lies packaged inside the nucleus in a highly complicated structure involving chromatin fiber, nucleoproteins, and repeating nucleosome arrays. A nucleosome is the fundamental chromatin unit, which consists of around 146 base pair units of DNA coated around a histone octamer that contains two copies each of the core histones H2A, H2B, H3, and H4 (Harshman et al., 2013; Misteli et al., 2000). Linker histones belong to the lysine-rich protein family responsible for DNA compaction and are located at the base of a nucleosome adjacent to the DNA entry/exit site to regulate the higher-order chromatin structure (Catez et al., 2006; Healton et al., 2020). Each linker histone H1 subtype has the same tripartite structure in higher eukaryotes, which contains one short N-terminal tail, a central globular domain, and a long disordered and highly basic C-terminal tail (Allan et al., 1980; Izzo et al., 2008; Talbert and Henikoff., 2021). Among them, the function of the C-terminal and the globular domain is to bind H1 to the nucleosome and to maintain the compact higher-order (30-nm) chromatin structure (Fyodorov et al., 2018; Hendzel et al., 2004; Robinson and Rhodes, 2006). Since the N-terminal domain is rich in alanine and proline, as well as other hydrophobic amino acids, it does not have a high affinity for DNA. Besides, both the N-terminal tail and the globular domain in linker histone H1 may undergo diverse post-translational modifications (PTMs), which have key functions in modulating the function and structure of chromatin (Fyodorov et al., 2018).

**FIGURE 1:**
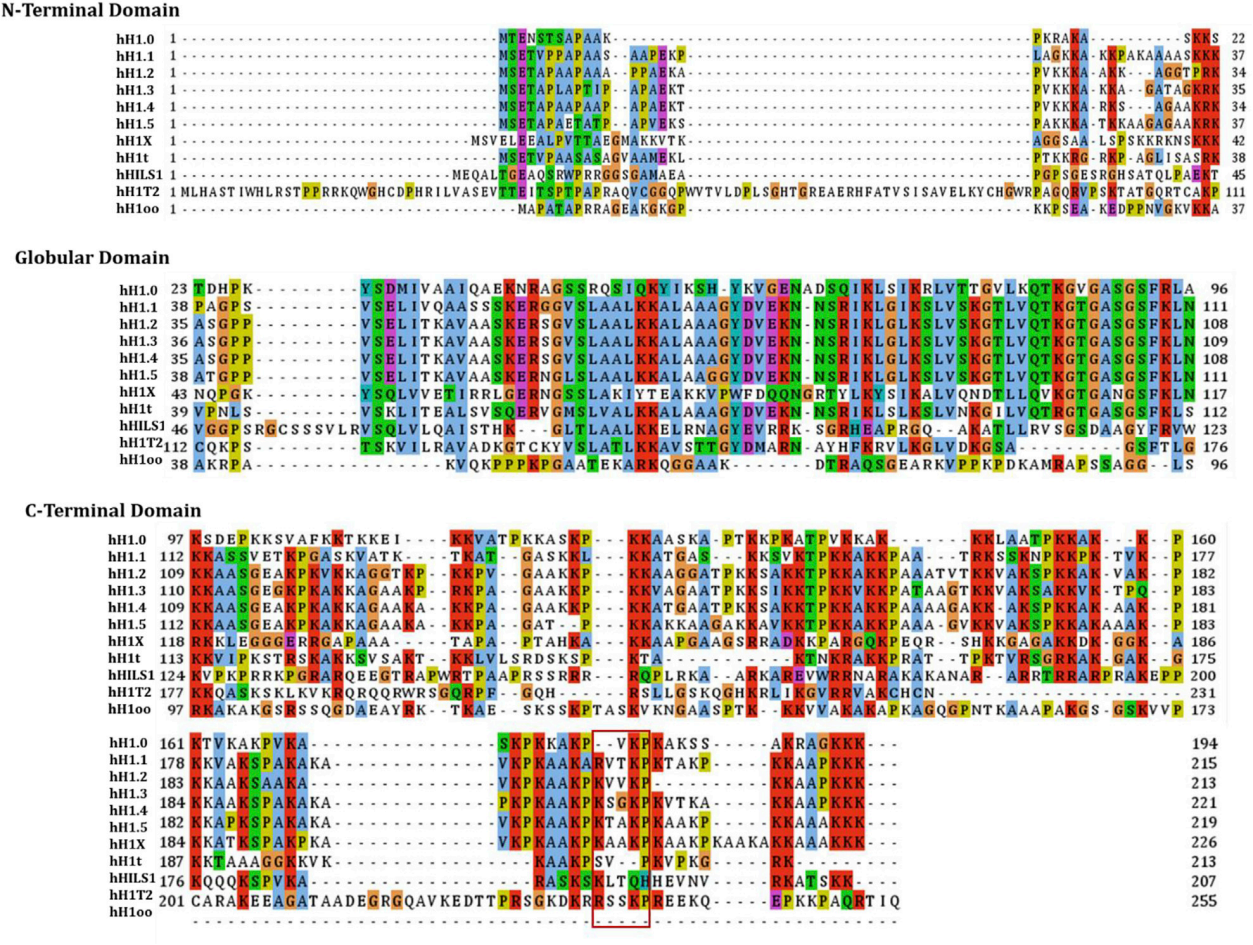
The sequence alignment of the human linker histones H1 family. Sequence alignment of 11 variants of human histone H1 were carried out by using Jalview, and the individual histone H1 variants sequences were downloaded from NCBI. The globular domain is highest conservation and the C-terminal tail is lowest conservation, and the K/RVVKP motif has only a low conservation amongst H1.1-H1.5 and HILS1.

The linker histone H1 family includes 11 variants in mammalian cells (Ausio., 2006; Happel snd Doenecke., 2009) (of which seven somatic subtypes (H1X, H1.0-H1.5) show differential expression within somatic cells), three testis-specific subtypes (H1t, HILS1, and H1T2), and one oocyte-specific subtype (H1oo) (Doenecke et al., 1997; Talbert et al., 2012; Tanaka et al., 2001). As shown in Figure 1, through the sequence alignment of 11 variants of human histone H1, the globular domain is highest conservation and the C-terminal tail is lowest conservation, and we have also found that the K/RVVKP motif has only a low conservation amongst H1.1-H1.5 and HILS1. As demonstrated in numerous studies, the different variants have different functions within various cells or specific cell processes, such as chromatin binding affinity, regulation of gene expression, knockout phenotypes, and interaction with specific partners (Clausell et al., 2009; Eirín-López et al., 2004; Millán-Ariño et al., 2014, 2016; Orrego et al., 2007; Ponte et al., 1998; Vyas and Brown, 2012). Among the somatic histone H1 variants, the linker histone H1.2 has been extensively detected within numerous tissues and cells (Meergans et al., 1997; Parseghian and Hamkalo., 2001) and is the most conserved of all histone H1 somatic variants. This implies that H1.2 may be evolutionarily significant. There is accumulating evidence that proves that the linker histone H1.2 has several important functions in multiple cellular processes, including apoptosis, autophagy, cell cycle control, and gene transcription. Most importantly, the effect of linker histone H1.2 on the response to DNA damage has attracted attention because H1.2 is involved in the interaction of factors related to DNA damage response and DNA repair machinery components. The linker histone H1.2 also seems to be related to the pathway of tumorigenesis and several other diseases, although the relation of linker histone H1.2 to such disorders remains largely unclear.

The present review discusses the latest advancements in the knowledge of linker histone H1.2’s effect on the response to and repair after DNA damage, as well as its effect on the regulation of gene expression and cell cycle. Additionally, its relations with some common disorders are also analyzed to shed more light on possible new therapeutic targets.

## H1.2 and DNA Damage Repair

Different endogenous or exogenous agents (ionizing radiation, chemicals) constantly attack the genome, which can result in DNA damage. In response to these threats, the eukaryotic cells have evolved repair mechanisms for DNA, recruiting factors related to DNA repair into the specific DNA damage sites, activating cell cycle checkpoints for preventing progression of the cell cycle, and finally mediating DNA damage repair (Ciccia and Elledge, 2010). It is only due to this elaborate DNA damage response pathway that cells can preserve the integrity of the genetic information inside them while also avoiding disease development and oncogenic transformation (Mills et al., 2003; Misteli and Soutoglou, 2009). The repair of DNA damage is carried out on chromatin with a high degree of structure. For efficient DNA repair, de-condensation of chromatin structure is necessary to allow repair factors to reach the site of DNA breaks (Papamichos-Chronakis and Peterson, 2013; Ziv et al., 2006). Members of the linker histone H1 family can combine with the nucleosome adjacent to the linker DNA entry/exit site, thus contributing to DNA stabilization and promoting higher-order chromatin compaction and folding (Woodcock et al., 2006). It has been shown that depletion of linker histone H1 family leads to profound alterations to chromatin structure, such as lowered core histone modifications, decreased local chromatin compaction, and reduced global nucleosome spacing (Fan et al., 2005). Additionally, it is known that linker histone H1 family deletion endows cells with resistance to DNA damage and enhanced checkpoint response in comparison to wild-type cells (Murga et al., 2007). Hence, linker histone H1 family has a key function in chromatin structure and its dynamics. Upon DNA damage, histone H1 can undergo many post-translational modifications and get released from the nucleosome, allowing chromatin de-condensation and accelerated recruitment of DNA damage response factors into the DSB sites. Nonetheless, despite the localized chromatin de-condensation to facilitate repair, the global chromatin fibers become rapidly compacted to protect cells from further damage, and linker histones can better stabilize such compaction (Hamilton et al., 2011).

**FIGURE 2:**
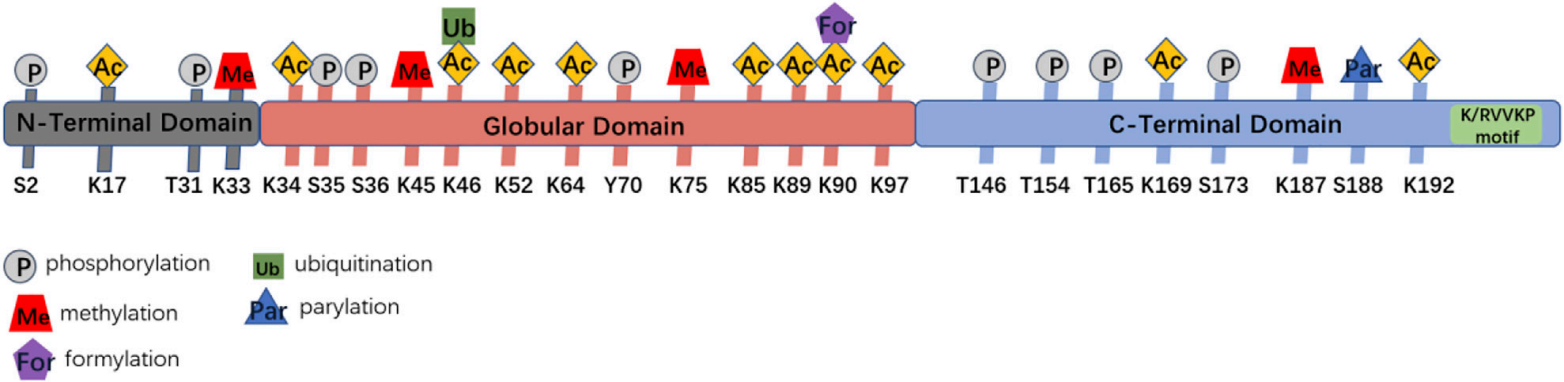
The scheme of functional domains and post-translational modifications sites of H1.2. H1.2 binds Bak *via* the C-terminal domain (V203) of K/RVVKP motif.

Linker histone H1 family has previously been proved to be one of the factors that facilitate DSB repair, and it is a part of the non-homologous end-joining (NHEJ) pathway (Rosidi et al., 2008), indicating that it is related to DNA repair. H1 is an important part of the chromatin fiber structure, which plays key roles in some chromatin-associated events, such as repair of DNA damage, although the precise mechanisms involved are yet to be elucidated. Experiments done to date reveal that linker histone H1.2, a subtype of H1, has a special function in regulating the response to and repair of DNA damage. As shown in Figure 2, several mass spectrometry studies have identified many kinds of modifications in the linker histone H1.2, such as phosphorylation, acetylation, ubiquitination, PARylation, formylation, and methylation (Jiang et al., 2007; Kouzarides., 2007; Wisniewski et al., 2007; Wisniewski et al., 2008; Weiss et al., 2010; Bonet-Costa et al., 2012; Sarg et al., 2015). Some of these modifications are related to DNA damage responses. Following DNA damage, H1.2 undergoes modifications such as phosphorylation by DNA-dependent protein kinase (DNA-PK) (Kim et al., 2012), ubiquitination by RNF8/RNF168 (Thorslund et al., 2015), and PARylation by PARP1 (Li et al., 2018b), that are critical for DNA repair (Table 1).

**TABLE 1:** The modification sites and function of H1.2.

Modification sites	Enzyme	Modification type	Function	References
T146	DNA-PK	Phosphorylation	Triggers p53-dependent DNA damage response; inhibits tumor cells growth and migration	Kim et al. (2012), Fan et al. (2019), Li et al. (2020)
T165	Unknown	Phosphorylation	A marker for proliferation and cancer malignancy	Terme et al. (2014)
Y70	FAK kinase	Phosphorylation	Plays a role in tumor cells proliferation	Perri et al. (2019)
K46	ITCH	Ubiquitination	Suppresses RNF8/RNF168-dependent formation of 53BP1 foci	Chang et al. (2019)
S188	PARP1	PARylation	Regulates ataxia telangiectasia mutated (ATM) activation	(Z. Li et al. (2018b))
K187	G9a and Glp1	Methylation	May make the nucleosome structure tighter	Weiss et al. (2010)

**FIGURE 3:**
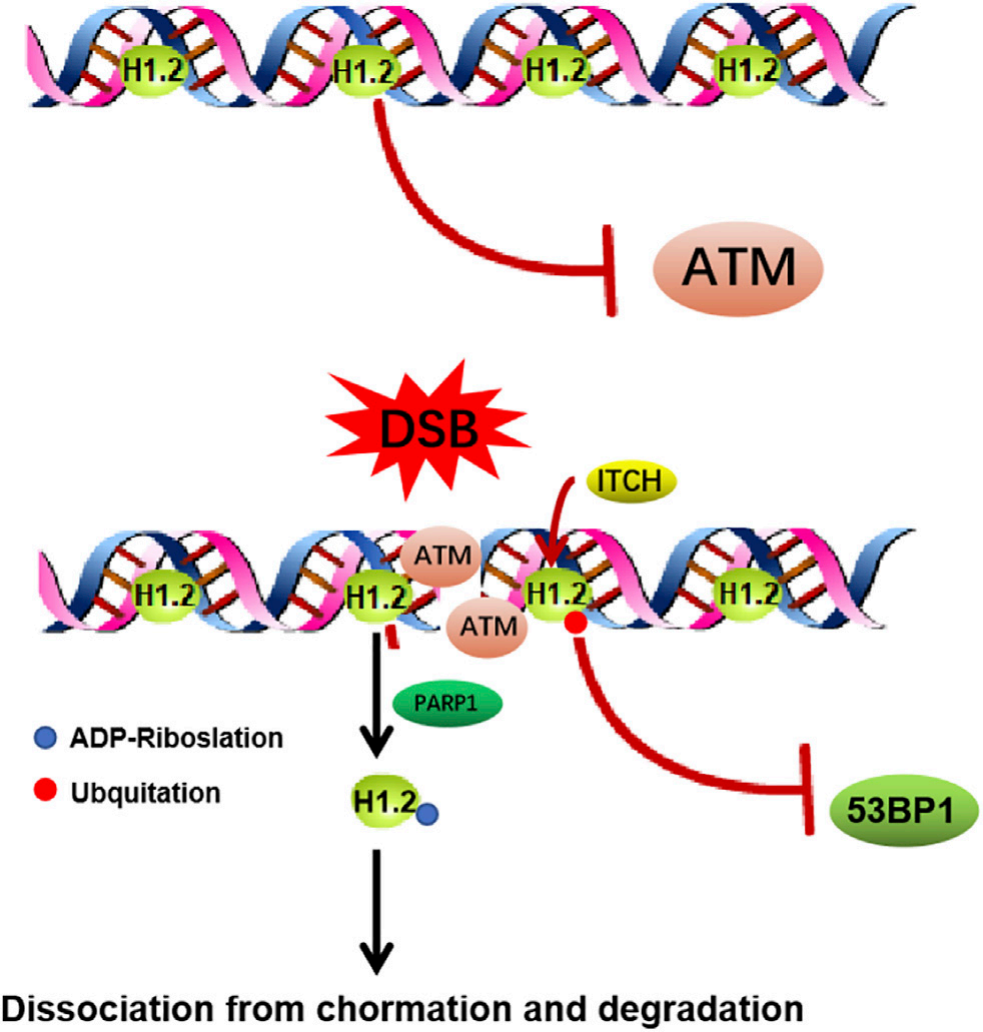
Functions of H1.2 in DNA double strand breaks repair. **(A)** H1.2 protects chromatin from aberrant ATM activation through direct interaction with the ATM HEAT repeat domain and inhibition of ATM (ataxia telangiectasia mutated) recruitment. Upon DNA damage, H1.2 undergoes rapid PARP1-dependent chromatin dissociation through PARylation (Protein poly ADP-ribosylation) and further proteasomal degradation, leading to ATM recuriment to sites of DNA damage and ATM activation, facilitating DNA damage repair. **(B)** ITCH-mediated polyubiquitination of H1.2 K46 site suppresses RNF8/RNF168-dependent formation of 53BP1 foci and DSB repair (ITCH: the specific E3 ubiquitin ligase).

Upon DNA damage, local changes to the condensation state of the chromatin are essential for the recruitment of the DNA damage repair factors. H1.2 has been shown to have a weaker ability to compact chromatin compared to most other variants, which endows H1.2 particular functions in DNA damage repair (Orrego et al., 2007; Prendergast and Reinberg., 2021). It has been shown that ITCH mediated H1.2 K46 ubiquitination suppresses DNA damage repair by impairing RNF8/RNF168-dependent formation of 53BP1 foci, which is a crucial component of NHEJ (non-homologous end joining) signaling (Chang et al., 2019). It also has been reported H1.2 inhibits HR (homologous recombination, HR) repair pathway through direct interaction with the ATM HEAT repeat domain and inhibition of MRE11-RAD50-NBS1 (MRN) complex-dependent ATM recruitment, which could be abolished by PARP1-dependent poly-ADP-ribosylation (PARylation) of its C terminus and further chromatin dissociation and proteasomal degradation (Li et al., 2018b). This study indicates that H1.2 is evicted from sites of DNA damage and H1.2 maintains chromatin stability while allowing for a less compact chromatin configuration so DNA repair enzymes can access the site of damage (Figure 3). Above all, it suggests us the possibility that un-modified H1.2 may inhibit HR by inhibiting HR repair factors foci formation, such as ATM. Once H1.2 been ubquited or PARylated, which changes the alkaline H1.2 to acidic, leading H1.2 easily dissociation from the acidic DNA, suppresses the NHEJ repair factor and facilitates HR.

The different H1 subtypes exert different functions at diverse stages during the repair of DNA damage. However, of all H1 subtypes, only H1.2 has been reported to specifically regulate DSB-induced apoptosis (Konishi et al., 2003). Linker histone H1.2, as a DNA packaging protein, not only functions in regulating gene transcription and chromatin structure but also in modulating the response to and repair of DNA damage. The following sections will discuss the post-translational modifications of H1.2 and their involvement in apoptosis after DNA damage.

## Role of H1.2 in Apoptosis and Autophagy

Apart from its functions within the nucleus, histone H1.2 also participates in DSB-induced apoptosis outside the nucleus (Gillespie and Vousden, 2003; Konishi et al., 2003; Yan and Shi, 2003; Norbury and Zhivotovsky, 2004; Zong, 2014). Upon x-ray irradiation or bleomycin-induced double-stranded breakage of DNA, but not UV irradiation, H1.2 is the only H1 subtype that has been reported undergoing a nuclear-to-mitochondrial translocation, where it accelerates the production of cytochrome c in isolated mitochondria in a Bak-dependent manner, which is a pro-apoptotic protein from the Bcl-2 protein family (Konishi et al., 2003; Okamura et al., 2008). In addition, it is seen that H1.2 deletion leads to resistance against apoptosis in mice and tumor cells (Konishi et al., 2003). The proapoptotic H1.2 histone protein is also suggested to participate in the formation of a protein complex along with apoptotic protease activating factor 1 (Apaf-1), cytochrome c, and caspase-9 when it is irradiated under UV. This sheds light on the role of cytosolic H1.2 in regulating the formation of an apoptosome (Ruiz-Vela and Korsmeyer, 2007). Therefore, H1.2 may hold the function of the nuclear-to-cytoplasmic transmission of apoptotic signals upon genotoxic damage. However, the precise mechanisms of how these apoptogenic signals induce cytoplasmic/mitochondrial redistribution of nuclear proteins and the specific apoptotic pathways involved remain poorly understood.

Earlier studies have demonstrated that the binding of H1.2 to chromatin is weaker than that of other histone H1 subtypes. Moreover, it has a weak chromatin compaction properties (Flanagan et al., 2016; Khadake and Rao, 1995; Orrego et al., 2007; Th’Ng et al., 2005) and weaker chromatin binding affinity that facilitate its rapid dissociation from DNA. This property may make histone H1.2 a very sensitive probe for the DNA double-strand breaks. H1.2 is one of the early nuclear intermediates that leak into the cytoplasm from the nucleus and function as a signaling molecule to initiate apoptosis.

It is also possible that post-translational modification of H1.2 may be associated with its redistribution. Several recent studies have suggested that after DNA damage, post-translational modifications of H1.2 such as PARylation and ubiquitylation occur, and these lead to H1.2 being more loosely bound to chromatin, thereby helping in its easy dissociation from chromatin (Li et al., 2018b; Thorslund et al., 2015). In addition, Konishi et al. have suggested that H1.2, together with some other histone H1 subtypes, showed p53-dependent nuclear-to-cytosol translocation (Konishi et al., 2003). Upon DNA damage, p53 could facilitate the recruitment of the histone acetyltransferase p300 (Espinosa and Emerson, 2001). But acetylation of p53 induced by p300 and H1.2 phosphorylation induced by DNA-PK, damage the interaction between p53 and H1.2 (Kim et al., 2012). Both modifications possibly facilitate the dissociation of H1.2 from chromatin. It is worth noting that histone H1 does not exhibit unique modifications under X-ray irradiation, with the same nuclear and cytosolic H1.2 forms (Konishi et al., 2003). Thus, whether post-translational modification of H1.2 or p53 has any function in H1.2 redistribution into the cytoplasm/mitochondria remains questionable.

Another possibility is that Bax/Bak, which are proapoptotic protein belongs to the Bcl-2 family, may regulate histone H1.2 and nucleophosmin redistribution and that such redistribution would not be related to Bax/Bak exposure at N-terminal, nor will it be suppressed by the overexpression of Bcl-xL (Lindenboim et al., 2010). According to such results, Bak and Bax act upstream to nucleophosmin and H1.2, which activate the latter by regulating their activities (Lindenboim et al., 2011). However, it has not yet been determined how the Bax and Bak regulate nuclear/cytoplasmic transport and redistribution from the respective action sites. It is also not known if there are other unknown apoptosis signaling pathways. Embryonic fibroblasts from untreated Bax/Bak double knockout mice showed a lower yet noticeable ratio of histone H1.2 nuclear/cytoplasmic redistribution, while staurosporine-treated or transfection-induced stresses triggered a moderate redistribution effect (Lindenboim et al., 2010). Hence, the redistribution of nuclear proteins may take place in a Bax/Bak-independent manner. However, the regulatory mechanisms underlying such effects remain largely unclear and deserve more investigation.

**FIGURE 4:**
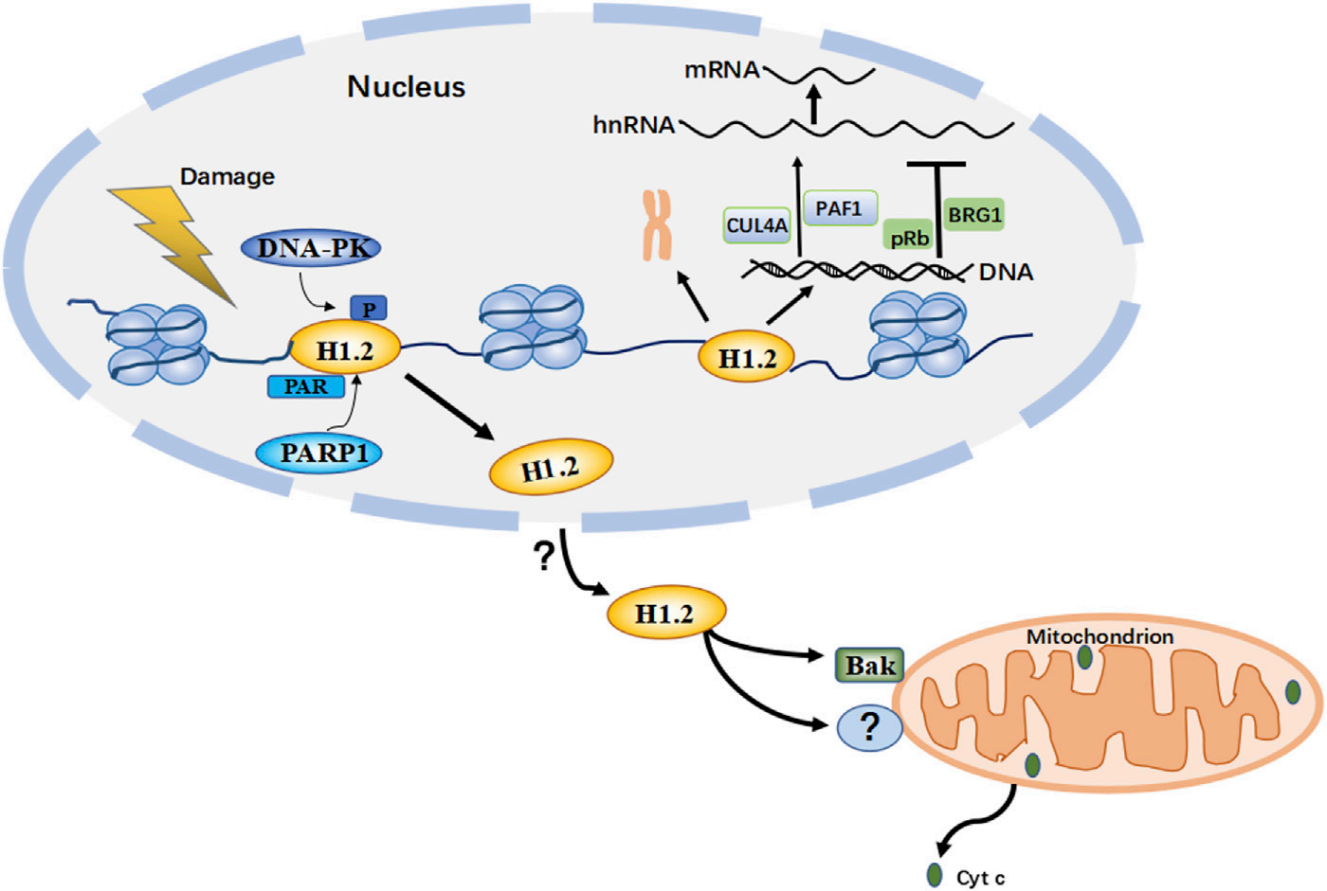
This is a hypothetical model based on multiple studies of H1.2 PTM (post-translational modifications) after DNA damage. When H1299 or U2OS cells are treated with DNA damage inducing agents etoposide or bleomycin, H1.2 is phosphorylated by DNA-PK (DNA-dependent protein kinase) in T146, these modifications allow the recruitment of transcription factors to the target promoter regions of p53 and the remodeling of chromatin in response to DNA damage; treating HeLa, U2OS or other tumor cells with etoposide or ionizing radiation, but not UV irradiation, H1.2 is PARylated (Protein poly ADP-ribosylation) in S188 by PARP1 (Poly (ADP-Ribose) Polymerase 1) and displaced from chromatin for degradation, which is necessary to appropriately activate ATM (ataxia telangiectasia mutated) for regulating the response to and repair of DNA damage; These modifications alter the binding affinity of H1.2 to chromatin, thereby dissociating H1.2 from chromatin. Upon DNA damage induced by bleomycin or X-ray in tumor cells such as MEFs and Hela, but not UV irradiation, *via* an unknown mechanism H1.2 then undergoes nuclear-to-mitochondrial translocation and activates Bak (is a pro-apoptotic protein from the Bcl-2 protein family) or other unknown proteins to mediate apoptosis through the mitochondrial pathway. H1.2 also functions to compact chromatin and regulate transcriptional activity within the nucleus. (Cyc c: cytochrome c.)

To better understand histone H1.2’s role in the mitochondrial apoptosis pathway and the underlying mechanisms, it is necessary to focus on its unique domains that are critical for its mitochondrial activity. Typically, its C-terminal domain plays an essential role in the apoptogenic activity, which regulates mitochondrial pathway apoptosis by antagonizing anti-apoptotic proteins (Garg et al., 2014). Consistent with this, another recent study suggests that histone H1.2 facilitates the direct activation of Bak *via* the C-terminal domain K/RVVKP motif while also simultaneously promoting the *in vitro* production of cytochrome c from mitochondria independent of the mitochondrial permeability transition (Schnetler et al., 2020). On the other hand, H1.2 does not contain a BH3 domain and is not significantly homologous to proteins belonging to the Bcl-2 family (Konishi et al., 2003). This suggests that Bak may be activated by different mechanisms, which remain to be elucidated. Interestingly, several nickel-affinity pull-down experiments have shown that H1.2 binds to Bak with a weak affinity (Schnetler et al., 2020), raising the question as to whether there are other sequence motifs responsible for the proapoptotic activity of H1.2. H1.2 function in the mitochondrion is dependent on other proteins such as the mitochondrial fusion protein Mfn-1 and the BH3 domain-only protein PUMA (Garg et al., 2014). This leads us to several questions: Is it possible for H1.2 to act on other mitochondrial components to regulate the apoptotic cascade? Do those nuclear/cytoplasmic redistribution mechanisms together with the specific apoptotic pathways need further investigation? (Figure 4).

Linker histone H1.2 can also function to regulate autophagy. Overexpression of histone H1.2 up-regulates the histone deacetylases HDAC1 and SIRT1 to maintain H4K16 de-acetylation, leading to the upregulation of autophagy related proteins, thereby promoting autophagy, inflammation, and lesion formation during early diabetic retinopathy (DR) (Wang et al., 2017). Such results suggest the potential of histone H1.2 as a new therapeutic target to prevent or treatment DR. However, how does histone H1.2 regulate the levels of HDAC1 and SIRT1, whether gene transcription inhibitor function of H1 histones relate to its regulation of HDAC1, SIRT1 levels and autophagy-related functions remains further investigation.

## H1.2 Regulates Gene Transcription and the Cell Cycle

All H1 subtypes (H1, H1.2-H1.5) are expressed in a replication- and S-phase dependent manner (Grunwald et al., 1991; Meergans et al., 1997). It has been reported the Gl/S-phase-block HeLa cells (0 h) are only a low histone H1 mRNA level, which is increased upon proceeding into S-phase and shows a drastic decrease in the process of transition from S-phase to G2/M (Meergans et al., 1997). This indicates H1 subtypes may facilitate the transition from G1 to S phase, genomic DNA replication in S phase, and inhibite the process of transition from S-phase to G2/M.

Among all H1 subtypes, H1.2 participates in cell cycle progression by regulating the expression of certain genes. H1.2 depletion is seen to reduce the levels of several proteins essential to the cell cycle, including CDK2 (Cyclin-Dependent Kinase 2), MCM2 (Minichromosome Maintenance Complex Component 2), and PCNA (Proliferating Cell Nuclear Antigen), causing G1 phase arrest in the cell cycle of human breast cancer (BC) T47D cells (Sancho et al., 2008). Moreover, H1.2 depletion inhibits cell proliferation mediated by progesterone receptor isoforms A (PRA) and promotes G2/M and S phase entry of the cell cycle (Pateetin et al., 2020). But it is still unclear whether H1.2 regulation of cell cycle depends on its expression changes during the cell cycle phases.

Linker histone H1.2 also plays a significant role in transcriptional activation. H1.2 can stably interacte with RNA polymerase II-associated factor 1 (PAF1) and E3 ubiquitin ligase cullin 4A (CUL4A) elongation complexes and can act as a transcriptional co-activator, regulating the post-translational modification of histone H4K31, and then recruiting RNA polymerase II into their respective promoter regions to activate the transcription of specific genes (Kim et al., 2013). Hence, it appears that linker histone H1.2 can activate specific genes.

However, despite its role in transcriptional activation, linker histones H1.2 are generally regarded as gene transcription inhibitors as they suppress transcription factors from accessing their corresponding sites within the chromatin and accelerating higher-order chromatin compaction (Kim et al., 2015; Misteli et al., 2000). The H1.2 protein has been suggested to significantly inhibit chromatin transcription mediated by p300 in a p53-dependent manner (Kim et al., 2008). For the H1.2 complex, its above-mentioned function involves its direct interaction with p53. This interaction can suppress chromatin acetylation mediated by p300. The expression of H1.2 significantly increases in lamina-related domains with abundant silenced genes and chromosomal domains with decreased guanine-cytosine (GC) levels (Millán-Ariño et al., 2014). H1.2 also interacts with BRG1, the chromatin remodeling complex, in an ATP-dependent manner, resulting in chromatin compaction and gene repression (Nacht et al., 2016). Yet another study has demonstrated that H1.2 and retinoblastoma tumor suppressor protein pRb interact with each other in the chromatin, which promotes pRb binding to the promoter regions of E2F’s target gene. H1.2 promotes transcriptional suppression in a pRb-dependent manner while facilitating cell cycle arrest depending on pRb. Meanwhile, in an environment good for cell growth, pRb can get phosphorylated by cyclin-CDK, as a result of which, the pRb-H1.2 complex dissociates from the chromatin. Following this, E2F can then activate the transcription of genes related to the cell cycle, and thus promote cell cycle progression (Munro et al., 2017; Munro and La Thangue, 2017). This study suggests that H1.2 has an important role in chromatin binding and inactivation of transcription and supress cell cycle in a pRb-dependent manner.

Additionally, post-translational modifications (PTMs) of H1.2 can modulate the transcription of specific genes. The PTM of H1.2 could impair the interaction of H1.2 with certain genes or lead to the dissociation of H1.2 from the chromatin, thereby attenuating its suppressive effects on genes transactivation (Kim et al., 2012; Li Z. et al., 2018). Consequently, it appears that linker histone H1.2 has dynamic functions specific to certain genes. Its specific function and its impact on gene transcription need to be further confirmed.

## Post-translational Modifications of H1.2

The different kinds of PTMs of H1.2 are related to chromatin remodeling, which can, in turn, regulate cell differentiation, gene expression, and DNA damage responses (Fan et al., 2005; Lawrence et al., 2015).

### Phosphorylation

H1 phosphorylation is the initial step for inducing chromatin remodeling, which allows recruiting of factors to activate specific genes and replicate the DNA. Site-specific phosphorylation at the C terminus of H1.2 is involved in a decondensation process which facilitates DNA replication (Talasz et al., 2009). Several phosphorylation sites of H1.2 (such as T31, T146, T154, T165, and S173 sites) have been identified in different cell lines, and these phosphorylations are enriched in the nucleoli (Garcia et al., 2004; Terme et al., 2014; Zheng et al., 2010). Notably, phosphorylation at S173 of histone H1.2 significantly increases during M phase relative to S phase, indicating that this event is cell cycle-dependent and may serve as marker for proliferation (Chen et al., 2016). H1.2 phosphorylation may be related to DNA damage repair, as the H1 phosphorylation status is used to indicate the genome-sustained cell damage level (Chubb and Rea, 2010). It has been proposed that the phosphorylation of human H1.2 promotes the response to DNA damage in a p53-dependent manner. Upon DNA damage induced by etoposide or bleomycin, H1.2 phosphorylated at the C-terminal domain T146 site by DNA-PK, and p53 acetylated by p300, which promotes the transcriptional activity of p53 by suppressing the interaction between p53 and H1.2. This promotes its suppression of the transactivation of p53. These modifications allow the recruitment of transcription factors to the target promoter regions of p53 and the remodeling of chromatin in response to DNA damage (Kim et al., 2012).

### Ubiquitination

The ubiquitin-dependent DNA damage signal transduction pathway accounts for a key mechanism in regulating DNA damage response (Jackson and Durocher, 2013). Ubiquitylation of H1.2 is suggested to be an important intermediate step for DSB repair mediated by the E3 ubiquitin ligases RNF168 and RNF8 (Huen and Chen, 2016; Thorslund et al., 2015). DNA double-strand breaks trigger non-proteolytic ubiquitylation (K63-linked) proteins in adjacent chromatin areas to generate binding sites for DNA repair factors. Two E3 ubiquitin ligases, RNF8 and RNF168, and UBC13, an E2 ubiquitin-conjugating enzyme that specifically generates K63-linked ubiquitin chains, are involved in this process. It has been described that RNF8 mediates K63 ubiquitylation at the ionizing radiation (IR) -induced DSB sites in an UBC13-dependent manner, and linker histones H1 represent major RNF8-UBC13-ubiquitylated chromatin substrates (Thorslund et al., 2015). Ubiquitylated forms of H1 function as the starting platform to bind RNF168; thus, triggering DSB repair factors recruitment. Moreover, the DSB-associated K63 ubiquitylation of H1 isoforms (H1.2 and H1x) was markedly up-regulated after DSBs, weakening the H1 proteins bounding to chromatin. In line with this, it has been reported that upon UV-mediated DNA damage, multiple sites of histone H1.2 are ubiquitylated by the E3-ligase HUWE1, H1.2-ub serves as a substrate for RNF8-UBC13-mediated K63-linked ubiquitylation, thereby stimulating the RNF8-RNF168 mediated DDR (Mandemaker et al., 2017). Such results suggest that H1.2 ubiquitylation possibly exerts a key effect on promoting chromatin remodeling, thus allowing for effective repair and serving as the mask to identify factors related to genome stability maintenance. However, despite its role in facilitates DNA damage repair, poly-Ubn of H1.2 may also suppress cellular DDR signaling. A recent data presented by Chang et al. (2019) suggests that the linker histone H1.2 K46 site can be efficiently and specifically ubiquitinated by ITCH, the specific E3 ubiquitin ligase, thereby suppressing 53BP1 locus formation depending on RNF8/RNF168. Such loci have key functions in response to (IR) -induced DNA damage. Poly-Ubn of H1.2 by nuclear AKT-activated ITCH suppresses cellular DDR signaling to counteract replication stress in cells. Consequently, H1.2 may act as a key signaling intermediate in the ubiquitin-driven DNA damage signal transduction cascade.

### ADP Ribosylation

H1.2 PARylation within S188 is related to the response to DNA damage, a novel mechanism proposed by Z. Li et al. (2018b) who suggested that H1.2 binds to chromatin when there is no DNA damage and blocks the interactions of ATM (ataxia telangiectasia mutated) with MRE11-RAD50-NBS1 (MRN) complex. ATM is a master kinase involved in the DNA damage response and repair, which phosphorylates several key proteins that initiate activation of the DNA damage checkpoint. It has been shown that H1.2 directly interacts with the ATM HEAT repeat domain, which leads to impaired ATM interaction with MRN or its substrates. This interaction was specific, as other H1 subtypes exhibited a much weaker binding affinity to ATM than H1.2 (Andrés et al., 2020). In contrast, when cells are treated with DNA damage agents etoposide or IR, but not UV irradiation, the C-terminal domain of H1.2 is poly-ADP-ribosylated (PARylated) by PARP1 and is displaced from chromatin for degradation, due to which ATM is replenished and activated *via* the MRN complex. Thereafter, ATM phosphorylates some protein substrates to activate DNA damage checkpoints. This study revealed that PARylation of H1.2 is necessary to appropriately activate ATM for regulating the response to and repair of DNA damage. PARP3 has also been shown to PARylate H1.2, but the mechanistic details remain further investigated (Rulten et al., 2011).

### Methylation

Lysine methylation has been discovered in H1.2. As demonstrated by Weiss et al. (2010), the two histone lysine methyltransferases G9a and Glp1 are responsible for methylating K187 in H1.2 *in vivo* and *in vitro*, respectively. Additionally, the G9a/Glp1-induced K187 methylation is specific to the H1 variant, which targets the H1.2 C-terminal domain with a stable methylation degree in the whole cell cycle. Methylation of H1.2 may make the nucleosome structure tighter (Liu et al., 2017), but the overall effect remains unclear.

PTMs of H1.2 have key functions in modulating chromatin response and DNA damage repair, thus guaranteeing cell homeostasis and genome integrity. Apart from phosphorylation, ubiquitination, PARylation, and methylation of linker histone, formylation, and acetylation have also been identified (Jiang et al., 2007; Li Y. et al., 2018; Wisniewski et al., 2008), although its effect has not been reported. PTMs generally lower the affinity of H1.2 for chromatin, thereby facilitating its dissociation. This allows for chromatin remodeling and also as an intermediate signaling step to regulate downstream functional effects in response to DNA damage (Figure 4). PTMs are related to the modulation of different H1 activities, but the underlying mechanisms are still unclear. Therefore, it is essential to systemically characterize H1.2 modifications to provide more insights into its effects on DNA damage response, modulation of gene expression, and the structure and function of higher-order chromatin.

## H1.2 Associated With Disease

As suggested by a series of studies, H1.2 has a key role in a variety of diseases, participating in tumorigenesis, autoimmune diseases, and viral infections. H1.2 is also involved in tumor growth, and high expression of H1.2 can predict poor outcomes for patients with pancreatic cancer (Song et al., 2008; Zhou et al., 2020). High expression of H1.2 was also found in some other cancer cells, and it has been reported that H1.2 can bind to H3K27me3, thereby inhibiting the expression of growth-suppressive genes and promoting the growth of cancer cells (Kim et al., 2015). Moreover, this process is dependent on the reciprocal binding of the C-terminal domain in H1.2 to H3K27me3. Thus, it can be implicated that H1.2 can affect the development of cancer cells by regulating the transcriptional activity of certain cancer-related genes.

The phosphorylation of H1.2 is also related to tumor cell growth. It is previously proposed that phosphorylation of H1.2 at the T146 position by DNA-PK impairs the interaction between p53 and H1.2. This decreases its suppression of the transactivation of the tumor suppressor p53, and ultimately inhibits tumor cell growth and promotes apoptosis (Kim et al., 2012). H1.2 phosphorylation at position T146 is potentially useful for bladder cancer screening, which may be used as a marker to predict disease invasion, relapse, progression, or therapeutic response (Telu et al., 2013). By regulating the degradation of the proteasome in DNA-PK, bladder cancer (BC) cell proliferation and invasion can be enhanced (Fan et al., 2019). In line with this, it has also been suggested recently that H1.2T146 phosphorylation mediated by DNA-PK can promote binding to Metastasis-associated 1 (MTA1, which shows over-expression within some human cancers) and inhibits pre-metastasis, MTA1-mediated cell proliferation, and invasion (Li et al., 2020). However, proteomic analysis in BC cells revealed the new tyrosine phosphorylation site Y70 in H1.2. Interestingly, this modification can be detected in the globular domain with a high conservation degree, and the level of tyrosine phosphorylation remarkably increases in BC cells in comparison to healthy cells, which indicates that such modification has a certain function in BC (Perri et al., 2019). Whether phosphorylation of H1.2 inhibits or promotes the growth of tumor cells may be related to different phosphorylation sites. But what can be concluded is that the phosphorylation status of H1.2 correlates with disease progression, which may serve as a biomarker for cancer.

H1.2 is also related to innate immunity, which can regulate the expression of viral genes (Conn et al., 2008). H1.2 is known to interact with influenza viral NS2 protein through its C-terminal and inhibits viral replication (Liu et al., 2017). Linker histone H1.2 is essential to inhibit virus replication and may be adopted as a candidate therapeutic target.

## Conclusions and Perspectives

H1.2 is known to be a nucleosome-binding protein regulating the structure of higher-order chromatin. More and more studies have presented several important characteristics of the linker histone H1.2 and its multiple functions. In this review, we have highlighted several studies that have indicated the effects of H1.2 on DNA damage response, the regulation of gene transcription, cell cycle, and its relationship with disorders.

PTMs of H1.2 act in a coordinated and orderly manner to regulate cellular events like repair of DNA damage, development, gene transcription, and progression of many types of tumor cells. More importantly, several modifications of H1.2 in different signaling pathways after DNA damage have been identified even though the specific underlying mechanisms remain unclear and the role of many other PTMs remains unknown.

In the future, more effort needs to be focused on determining H1.2-mediated protein-protein interactions, as well as on mapping the domains responsible and identifying the mechanisms through which they act. This will lead to clarification of the many gaps in our current understanding of the molecular basis for the multifunctional nature of the linker histone.

H1.2 interacts with many different DNA damage involved proteins making its role in DNA damage highly complex. Given several studies show H1.2 dissociated from chromatin after DNA damage, perhaps this process allows for a less compact chromatin configuration so DNA repair factors can access the site of damage. Several studies published recently have described the important role of H1.2 in DNA damage repair. H1.2 regulates both HR factor (ATM) and NHEJ factor (53BP1) recruitment to sites of DNA damage, it indicates H1.2 may participates in the DSB repair pathway choices. And, it remains unclear whether H1.2 facilitates or represses DNA damage repair, which needs to be further investigation.

H1.2 is the only isoform of linker histone H1 family that has been reported to disassociated from nuclear and transport to cytoplasm under several stimulations, which is essential for its cytoplasmic function, such as autophagy and apoptotic. Although it has been reported ubquitation and PARP1 mediated PARlation regulates H1.2 dissociation from chromatin and translation to cytoplasm, it remains unclear which modification and which sites on H1.2 directly determines this process.

Even though several studies have identified the role of H1.2 in cytochrome-c release and apoptotic, however, the results of our lab shows knocking-down H1.2 in HeLa and MD231 cells has faint effect on the percentage of apoptotic cells, even cells were treated with irradiation. How H1.2 mediates apoptotic and cytochrome-c release need further investigation. It may not be easy to answer these questions because some other proteins may also redistribute within the identical cell in case of apoptosis, and some of them may have apoptotic effects themselves, which makes it difficult to rule out other factors to study the role of H1.2 alone. Dissecting these pathways mechanistically is a challenge and provides room for subsequent research.

The H1.2 histone has a gene-specific and dynamic function in modulating gene transcription, and its altered expression has been associated with cancer. H1.2 also regulates the transcriptional activity of several cancer-related genes, thereby affecting cell proliferation as well as cancer development. Thus, H1.2 may serve as a candidate target for anti-cancer therapeutics. Hence, there is a need for further experiments to explore and illustrate the mechanisms governing these effects at the molecular level.
